# Locating perpetrators of violence against women in India: An analysis of married men’s characteristics associated with intimate partner violence

**DOI:** 10.1371/journal.pone.0289596

**Published:** 2023-08-04

**Authors:** Rakesh Chandra, Sonal Srivastava, Aditya Singh, Saradiya Mukherjee, Jeetendra Kumar Patel

**Affiliations:** 1 School of Health Systems Studies, Tata Institute of Social Science, Mumbai, India; 2 Independent Researcher, Mumbai, India; 3 Department of Geography, Banaras Hindu University, Varanasi, India; 4 Department of General & Applied Geography, Dr. Hari Singh Gour University, Sagar, India; Virtual University of Pakistan, PAKISTAN

## Abstract

Intimate Partner Violence (IPV) against married women is widely prevalent in India. Using recent data from NFHS-5, we analyzed the association between husbands’ characteristics and IPV. Separate logistic regression models were developed for three distinct “husband characteristic groups” namely demographic, social and economic groups, and one final model including only statistically significant variables. IPV has been found to be significantly associated with men’s age, age gap between husband and wife, men’s educational level, religion, caste, region, number of daughters, wife’s decision-making autonomy, men’s IPV justifying attitude, alcoholism and substance abuse among men, type of work and wealth. We suggest shifting the policy gaze from women and prioritizing men’s education, control on substance abuse and alcoholism among men as well as employment opportunities to tackle the violence against women.

## Introduction

Intimate Partner Violence (IPV) is considered to be a social scourge and serious public health concern. IPV is defined as all forms of physical, sexual and emotional abuse perpetrated against the intimate partner in a close relationship, including current and former spouses and dating partners [1]. It is recognized as a criminal offense in India and punishable under Section 498-A of the Indian Panel Code.

As per the most recent estimates, globally over a quarter of all ever-partnered women of reproductive age have experienced physical or sexual or both forms of IPV, which averages around 35% in south Asia [1]. In India, 32% of ever-married women reported having experienced physical, sexual, or emotional violence by their husbands in their lifetime. The most common type of spousal violence, in India, is physical (28%), followed by emotional (14%), and sexual (6%). While a recent pan-India survey reported a drop, from 31% to 29% (in a period of 5 years) in physical and sexual violence combined, nonetheless, the magnitude is still unacceptably high [2]. There is considerable evidence to suggest the spousal violence leads to poor health, injuries, malnutrition, pregnancy complications, risks of sexually transmitted infections and Human Immunodeficiency Virus (HIV). It leads to emotional stress, depression, anxiety, post-traumatic stress disorder (PTSD), sleep disorders and fear and an increased risk of alcohol and substance abuse [3–12]. Additionally, it was found in a study that 38–50% of all homicides of women are committed by their partners as a resultant of IPV [13].

In India, among all ever-married women who experienced domestic violence, 84% reported their current husband as the perpetrator [2]. IPV in India is deeply rooted in its patriarchal nature, stemming from cultural norms and a conservative social structure. It serves as a manifestation of the unequal power dynamics between men and women within marital relationships. The origins of IPV can be traced back to the concept of patriarchy, as elucidated by Freidrich Engels in his classic work *Origin of the Family*, *Private Property and the State*. Engels theorizes that with the advancement of agriculture, the need for private landholding arose, subsequently highlighting the importance of inheritance within societies. As a result, women began to be treated as objects of property whose primary role was to procreate and maintain the family lineage. The control exerted by men over women became a tool for upholding patriarchal dominance, leading to male supremacy within the household. Any perceived threat to this dominance often triggers incidents of domestic violence [14].

Men’s engagement in IPV can be understood through various theories on domestic violence, which can be classified into three main categories: socio-political critiques, psycho-analytical theories, social learning theories. Socio-political critique theories argue that domestic violence serves as a mechanism to uphold and protect society’s patriarchal and hierarchical social structure [15]. These theories highlight the role of power dynamics, gender inequality, and societal norms in perpetuating domestic violence. Interestingly, in families where women are employed and husbands earn less, it has been noted that the latter are more likely to engage in violence presumably to have an upper hand in the power dynamics of the household and control over the female partner [8, 16], such paradox can be associated with Status Inconsistency theories [17].

Psycho-analytical theories center around the impact of stress, anxiety, anger, and guilt experienced in a married life and during raising a child. According to these theories, men may exhibit hyper masculine behavior as a means to compensate for their emotional insecurities and stress. Factors such as impulsive nature, aggression, anti-social behavior, toxic masculinity, controlling tendencies, poor negotiation skills, low self-esteem, and insecurity have been associated with men who are more prone to subjecting their spouses to domestic violence [12, 18–20]. Among men, particularly the sole providers, increased drinking and substance abuse due to financial and other household stresses leads to destructive behavior, often inflicting violence on the others, usually the spouse [5, 20].

Social learning theories, following the works by Bandura, emphasize the intergenerational transmission of violence within families [21]. According to these theories, individuals acquire violent behavior through the process of observing and imitating it within their family environment [22]. The exposure of children to their parents’ physical aggression and intimate partner violence (IPV) contributes to the generational social learning of such behaviors and is associated with an increased tendency to justify IPV. Moreover, social learning theory suggests that male children learn aggression from their parents and tend to replicate this behavior in their own adult relationships [15].

The exiting theorization of violence on women has man at its core as preparator, however, in exiting research generally woman is found to be at the core of inquiry. Thus, the first step toward any solution requires a comprehensive understanding of men’s indulgence in IPV by identifying perpetrators through their bio-demographic, psychological and social characteristics. Several studies have established that in IPV cases, attitude towards wife-beating, socio-economic conditions, substance abuse, and controlling behaviors of husbands pose a greater risk of violence against wives [23–26]. IPV has also been linked to various bio-demographic, social, economic, and behavioral aspects of the perpetrators [27, 28]. Globally, a few most common characteristics of husbands perpetrating IPV are found to be rural residence, low educational and economic status, early marriage, alcohol, and substance abuse, justifying wife beating attitude and controlling behavior [5, 10, 28–30]. In Indian context, there are numerous studies addressing broad aspects of IPV, including early marriage [10, 31], physical and mental health of the victims [12], help-seeking options [32], the role of neighbor support [33], the relative status of men and women in IPV cases [16], gender inequality measures and socio-economic status [34], IPV amongst SC women [35], contraceptive use and IPV against women [36].

The existing literature on domestic and spousal violence tends to focus on women, the victims, neglecting the characteristics, perceptions, and attitudes of perpetrators, particularly in India. Understanding the root causes of intimate partner violence (IPV) in India requires a deeper understanding of the characteristics of those who commit such violence. Previous attempts to study IPV perpetrating husbands in India have been limited in scope, primarily consisting of small-scale qualitative studies that fail to provide a comprehensive empirical understanding and generalizations. The study also drives its relevance and significance from the fact that Sustainable Development Goals emphatically call for the elimination of gender-based violence against women and girls (target 5.2) [37]. In this study, therefore, we aim to comprehensively analyze Indian husbands’ characteristics and their association with indulgence in IPV. From policy perspectives, we aim to perform an empirical study of perpetrators of IPV and thus help shifting the policy gaze on them from victims for intervention and prevention of spousal violence.

## Data and methods

### Data source

The study uses the data from the fifth round of the National Family and Health Survey (NFHS-5) conducted during 2019–21. This is a nationally representative dataset is collected by the Ministry of Health and Family Welfare, Government of India, and the International Institute of Population Science (IIPS), Mumbai (India) [2]. NFHS-5 interviewed 7,24,115 women and 1,01,839 men with a response rate of 96.9% and 91.6%, respectively. This study used the ‘Couples Recode’ file to quantify responses from the currently married women experiencing IPV and their current husbands on their demographic, social, and economic background. A total of 47,918 women were selected for the Domestic Violence Module (DVM)—a section of NFHS-5 survey specially designed to capture information on IPV—out of which 1430 could not be interviewed due to privacy issues. A total of 46,488 women and their current husbands were interviewed for the DV module.

#### Ethics statement

The protocol for the NFHS-5 survey, was reviewed and approved by the International Institute of Population Sciences (IIPS) Institutional Review Board and the ICF International Institutional Review Board. An informed written consent was obtained from each of the participants in the survey. This study analyses the DVM module data from NFHS-5 survey, which is publicly accessible adhering the confidentiality and anonymity protocols outlined in the Helsinki declaration.

### Outcome variable

The outcome variable of this study, “Men’s indulgence in IPV”, has been created from the variable “whether or not the respondent woman has ever experienced spousal violence” in the dataset. The NFHS survey only asked women whether they have suffered any of the 13 acts of violence by their current husband, categorized under three distinct groups, i.e., physical, emotional, or sexual violence. The set of questions started with three questions related to acts of emotional violence, three related to sexual violence, and seven related to acts of physical violence. The NFHS-5 has also further classified each act of physical violence into less severe and severe categories. Since the data is taken from the “Couples Recode” data file which includes responses from current married partners only i.e. women and their current husbands. Therefore, women’s experience of IPV (by current partner) has been reframed as husband’s “indulgence in IPV” for the current study.

The women who have responded “Yes” to at least one of these questions were considered to have experienced IPV and therefore are taken as husband’s “indulgence in IPV” and coded “1”. Those who responded “No” to all questions were coded “0” as they were considered “not to have experienced IPV” of any form or their husband “not indulging in IPV”, in the outcome variable in the regression model.

### Independent variables

This study aims to explore the characteristics of Indian husbands perpetrating violence against their wives. The ’DV module’ of the NFHS survey only interviewed selected women about their experience of IPV. The ‘Couples Recode’ data file provides the data of ever-married women from the DV module merged with the variables on characteristics of husbands (as responded by themselves in “Man Questionnaire”) of such women (selected and interviewed for DVM). All the characteristics of men (husbands) were grouped under three categories i.e., (1) demographic, (2) social and (3) economic characteristics for further analysis and regression models. These variables are listed in the given S1 Table.

### Statistical analysis

Pearson’s chi-square tests were applied to examine the association of IPV with husband characteristics as a first step in the analysis. The analysis aimed to provide an overview of the statistical relevance of the range of husband’s characteristics variables in the context of their indulgence in IPV. It also helped us to eliminate the variables statistically not associated with the IPV. In the second step, unadjusted odds ratios were calculated for each independent variable (categorized in three "husband characteristics groups," i.e., social, demographic, and economic) included in the study. The unadjusted odds ratio helped us in recognizing the robustness and magnitude of the statistical relationship between each of the husband characteristics and IPV against women. It also helped in detecting the relevant variables (in each of the three husband characteristics groups) to be fitted in our final model. Only those predictor variables with statistically significant (p <0.05) unadjusted odds were included in the final logistic model. The logistic regression model results have been presented in the form of odds ratios with p-values and 95% confidence intervals. As the last step of the analysis, binary logistic regression model was fitted to describe the net effect of predictor variables on the outcome variable IPV. The logistic regression model fitted for the study is specified as below:

$$\log\left(\frac{\pi }{1-\pi }\right)=\beta_{0}+\beta_{1}X_{1}+\beta_{2}X_{2}+\ldots \ldots +\beta_{n}X_{n}$$

Where π indicates the probability of an event (here IPV), *β*_*0*_ is the y-intercept, *β*_*i*_ indicates the regression coefficients associated with the reference group, and *x*_*i*_ indicates the predictor variables. Stata 16 statistical software was used for analyzing the raw data [38].

## Results

About 30% of women in our study sample reported experiencing at least one type of violence among the 13 acts of physical, emotional, and sexual violence during the interview. Out of which largest proportion of women, about 26%, have reported having suffered physical violence from their husband, the most common form of which remains slapping, reported by about 77% of all IPV experiencing women.

The next most prevalent form of IPV is reported to be emotional violence experienced by about 12% of all married women followed by sexual violence by 5% of all married women. Amongst all IPV experiencing women, physical violence is reported by about 88%, the most common of all, followed by emotional violence by 39.56%, and sexual violence by 17.21%. Of those who experienced IPV of any kind, nearly 23% have suffered severe physical violence. 25% have been humiliated, and 22% have been insulted. The most common form of sexual violence is being physically forced into unwanted sex by the husband (Table 1).

**Table 1:** Prevalence of IPV among ever married women in India, NFHS-5 (2019–21).

Type of violence experienced by ever-married woman	No. of ever-married women who have experienced IPV (out of 46488)	%age of ever-married women who have experienced IPV (out of 46488)	%age of different types of violence among IPV experiencing women (out of 13922)
**Physical Violence**	**12,304**	**26.47**	**88.38**
Pushed/Shook/ had something thrown at	5042	10.84	36.21
Slapped	10,813	23.26	77.67
Punched with a fist or hit by something	2,855	6.14	20.50
Kicked or dragged	3,046	6.55	21.88
Been strangled or burnt	750	1.61	5.38
Been threatened with knife/gun or other weapon	469	1.01	3.37
Had arm twisted or hair pulled	4,084	8.79	29.33
*Less severe physical violence*	*12*,*066*	*25*.*96*	*86*.*67*
*Severe physical violence*	*3*,*247*	*6*.*98*	*23*.*32*
**Emotional Violence**	**5,508**	**11.85**	**39.56**
Humiliated	3,569	7.68	25.63
Threatened with harm	2,232	4.80	16.03
Insulted or made to feel bad	3,168	6.81	22.75
**Sexual Violence**	**2,396**	**5.15**	**17.21**
Physically forced into unwanted sex	1,787	3.84	12.83
Forced into other unwanted sexual acts	981	2.11	7.05
Physically forced to perform sexual acts respondent didn’t want to	1,372	2.95	9.85
**Overall experience of IPV**	13,922	29.95	

### IPV by husband’s characteristics

Table 2 presents the prevalence of IPV by the background characteristics of the victims’ husbands. Husbands who are old, illiterate, smoke (32%), consume tobacco (35%), or drink alcohol regularly (44%) have the highest tendency to commit IPV. The prevalence of husbands committing IPV is highest among Hindus (31.42%) and Scheduled caste (34%) population and in the southern region (38.5%) of the country. Economically, those who are poorest, employed in unorganized sectors, and indulge in heavy manual labor commit a higher number of violent acts on their wives. The prevalence of IPV is increasing with the increasing age of husbands. The highest proportion of IPV is committed by husbands older than 40; over 30% of them have committed violence against their wives. The age gap between husband and wife is found to be a protective factor where the prevalence of IPV decreases with an increase in age gap, where prevalence is highest when husbands are 5–10 years older than the wife (31%). The health risks of husbands related to abdominal obesity also play an important role in the capabilities of acts of IPV by husbands; the healthier, more fit men commit more IPV (30.5%).

**Table 2:** Prevalence of IPV by husband characteristics in India, NFHS-5 (2019–21).

Background variable	Total Number	Women who had experience IPV	Chi-square (Z)	P-value
		[13922 (29.95%)]		
***Demographic***				
**Husband’s age**			70.48	<0.001
15–19	57	15 (26.32)		
20–29	7410	1956 (26.40)		
30–39	18522	5499 (29.69)		
40–49	15737	4912 (31.21)		
>50	4762	1540 (32.34)		
**Age gap**			25.27	0.0001
Younger than wife	1662	412 (24.79)		
0–5	30070	9078 (30.19)		
5–10	11743	3562 (30.33)		
10–15	2558	747 (29.20)		
15–20	369	100 (27.10)		
>20	86	23 (26.74)		
**Abdominal obesity**			19.57	0.0001
Low	33827	10313 (30.49)		
Moderate	7723	2027 (27.97)		
High	3268	1582 (29.22)		
***Social***				
**Husband’s education**			786.58	<0.001
No education	7579	2986 (39.40)		
Primary	7116	2517 (35.37)		
Secondary	25301	7144 (28.24)		
Higher	6492	1275 (19.64)		
**Religion**			239.43	<0.001
Hindu	65367	11113 (31.42)		
Muslim	5413	1591 (29.39)		
Christian	3383	712 (21.05)		
Other	1819	506 (21.76)		
**Caste**			266.50	<0.001
SC	8701	2970 (34.13)		
ST	9454	2728 (28.86)		
OBC	17765	5662 (31.87)		
Other	8229	1941 (23.59)		
**Residence**			103.27	<0.001
Urban	11420	2988 (26.16)		
Rural	35068	10934 (31.18)		
**Region**			898.40	<0.001
Northern	8230	1657 (20.13)		
Central	10708	3512 (32.80)		
Eastern	7325	2588 (35.33)		
Western	5350	1332 (24.90)		
Southern	7521	2900 (38.56)		
North-eastern	7354	1933 (26.29)		
**Number of daughters**			142.47	<0.001
0	12673	4754 (27.28)		
1–2	17462	7781 (30.82)		
>2	2431	1387 (36.33)		
**Wife’s Decision Making autonomy**			171.40	<0.001
Low	4061	1469 (36.17)		
Medium	6813	2328 (34.17)		
High	35569	10116 (28.44)		
**IPV justifying attitude**			440.22	<0.001
No	28381	7489 (26.39)		
Yes	18107	6433 (35.53)		
**Smoking**			25.36	<0.001
No	38386	11307 (29.46)		
Yes	8102	2615 (32.28)		
**Consumes Tobacco**			373.46	<0.001
No	29948	8055 (26.90)		
Yes	16540	5867 (35.47)		
**Alcohol frequency**			457.18	<0.001
None	30946	8409 (27.17)		
Regularly	2779	1223 (44.01)		
Occasionally	12763	4290 (33.61)		
***Economic***				
**Labor Type**			344.52	<0.001
Unemployed	1237	336 (27.16)		
Mental work	2365	481 (20.34)		
Mental and Manual labor	3273	764 (23.34)		
Light Manual labor	9935	2665 (26.82)		
Heavy Manual labor	27592	9106 (33.00)		
**Wealth Index**			675.24s	<0.001
Poor	20566	7258 (35.29)		
Middle	9740	2974 (30.53)		
Rich	16182	3690 (22.80)		

The prevalence of IPV decreases with an increase in the husband’s educational level, highest amongst illiterates (40%) and twice as low (20%) amongst those who have attained higher education. Similarly, the rural dweller husbands commit more IPV than urban residents. A husband’s employment status and type of work directly affect the prevalence of IPV in married couples. The husbands employed in the unorganized sector have the highest ratio (32%) of perpetrating IPV as the pressure and insecurity that comes with unorganized labor often translates into frustration being unloaded on wives. Similarly, husbands with heavy manual labor are most violent (33%) to their wives.

The prevalence of IPV decreases as household wealth increases. Husbands of the “poor” (35.29%) wealth quintiles had the highest proportion of committing IPV, which gradually decreases in the subsequent quintiles, as low as 22% in the richest quintile.

### IPV and husband characteristics: Logistic regression models

Three separate logistic regression models incorporating all the variables from three “husband characteristics groups” were fitted with IPV as the outcome variable. These three models result in three sets of odds of IPV by husbands’ demographic, social and economic characteristics. A final regression model was fitted with all the statistically relevant variables for adjusted odds of IPV by husband characteristics. “Place of residence” turned out to be statistically insignificant at a 5% level in the collective model. Hence, it was removed from the final regression model. All selected variables except residence were found to be significantly associated with IPV committed by husbands (Table 3).

**Table 3:** Odds ratios with 95% CI of the relationship between lifetime IPV and husband characteristics in India, NFHS-5 (2019–21).

Background variable	Sub Group Models				Final Model			
	OR	95% CI		p-value & significance	OR	95% CI		p-value & significance
		Lower	Upper			Lower	Upper	
***Model 1 Demographic***								
**Husband’s Age**								
15–19	Ref							
20–29	1.727	0.955	3.122	0.071*	1.536	0.832	2.838	0.170
30–39	2.271	1.258	4.101	0.007***	1.923	1.042	3.548	0.037**
40–49	2.340	1.296	4.226	0.005***	1.869	1.012	3.451	0.046**
>50	2.367	1.308	4.283	0.004***	1.977	1.068	3.660	0.030**
**Age Gap**								
Younger than wife	Ref							
0–5	1.321	1.140	1.532	0.000***	1.116	0.948	1.315	0.187
5–10	1.280	1.101	1.489	0.001***	1.101	0.931	1.302	0.259
10–15	1.239	1.047	1.465	0.013**	1.032	0.856	1.245	0.740
15–20	0.910	0.686	1.206	0.512	0.683	0.496	0.939	0.019***
>20	0.897	0.555	1.449	0.657	0.586	0.329	1.044	0.070*
**Abdominal Obesity**								
Low	Ref							
Moderate	0.845	0.799	0.893	0.000***	0.863	0.812	0.918	0.000***
High	1.060	0.982	1.144	0.136	1.103	1.014	1.198	0.022**
***Model 2 Social***								
**Husband’s Education**								
No education	Ref							
Primary	0.854	0.794	0.919	0.000***	0.896	0.830	0.968	0.005***
Secondary	0.668	0.629	0.709	0.000***	0.747	0.699	0.798	0.000***
Higher	0.490	0.450	0.534	0.000***	0.581	0.525	0.643	0.000***
**Religion**								
Hindu	Ref							
Muslim	0.907	0.843	0.976	0.009***	0.954	0.881	1.033	0.242
Christian	0.648	0.557	0.752	0.000***	0.671	0.573	0.786	0.000***
Other	0.994	0.851	1.161	0.938	0.953	0.806	1.127	0.575
**Caste**								
Schedule Caste	Ref							
Schedule Tribe	0.913	0.840	0.992	0.031**	0.869	0.797	0.948	0.002***
OBC	0.899	0.849	0.952	0.000***	0.903	0.849	0.959	0.001***
Other	0.792	0.738	0.850	0.000***	0.819	0.759	0.883	0.000***
**Residence**								
Urban	Ref							
Rural	1.109	1.054	1.167	0.000***				
**Region**								
Northern	Ref							
Central	1.620	1.451	1.809	0.000***	1.521	1.355	1.708	0.000***
Eastern	1.741	1.574	1.924	0.000***	1.605	1.442	1.787	0.000***
Western	1.117	1.009	1.237	0.033**	1.094	0.983	1.218	0.100
Southern	2.000	1.805	2.216	0.000***	1.990	1.785	2.218	0.000***
North-eastern	1.486	1.296	1.704	0.000***	1.378	1.192	1.593	0.000***
**Number of Daughters**								
0	Ref							
1–2	1.094	1.043	1.148	0.000***	1.079	1.025	1.136	0.004***
>2	1.427	1.317	1.545	0.000***	1.348	1.236	1.470	0.000***
**Decision Making Autonomy**								
Low	Ref							
Medium	0.897	0.823	0.978	0.014**	0.942	0.860	1.033	0.202
High	0.835	0.775	0.900	0.000***	0.861	0.795	0.932	0.000***
**IPV justifying attitude**								
No	Ref							
Yes	1.211	1.155	1.269	0.000***	1.181	1.124	1.241	0.000***
**Smoking**								
No	Ref							
Yes	1.133	1.064	1.206	0.000***	1.123	1.051	1.200	0.001***
**Consumes Tobacco**								
No	Ref							
Yes	1.350	1.284	1.420	0.000***	1.312	1.244	1.383	0.000***
**Alcohol Frequency**								
Never	Ref							
Regularly	2.053	1.862	2.263	0.000***	2.020	1.822	2.239	0.000***
Occasionally	1.194	1.131	1.260	0.000***	1.220	1.153	1.291	0.000***
***Model 3 Economic***								
**Labor type**								
Unemployed	Ref							
Mental work	0.836	0.709	0.985	0.032**	0.930	0.776	1.115	0.432
Mental and Manual	0.652	0.559	0.760	0.000***	0.654	0.553	0.773	0.000***
Light Manual labor	0.759	0.661	0.872	0.000***	0.720	0.619	0.837	0.000***
Heavy Manual labor	0.962	0.841	1.100	0.569	0.821	0.710	0.950	0.008***
**Wealth Index**								
Poor	Ref							
Middle	0.815	0.771	0.861	0.000***	0.919	0.862	0.980	0.010***
Rich	0.531	0.504	0.559	0.000***	0.686	0.641	0.734	0.000***

Notes: Ref = Reference Category, OR = Odds Ratio, CI = Confidence Interval

***p <0.01

**p <0.05

*p <0.10.

#### Model 1 demographic characteristics

In this model, IPV was found to be positively associated with age of husbands. The men over 30 years are more than twice as likely to commit violence against their wives as compared to those aged below 20. The age gap between husband and wife also positively related to violence (OR ranging 1.321–1.239) although a decreasing trend can be observed as the age gap increases. Moderately obese husbands are 15% (OR = 0.845, 95% CI = 0.799–0.893) less likely to commit IPV than those with low obesity.

#### Model 2 social characteristics

In this model, all 10 social characteristics of husbands were found to be significantly associated with the occurrence of IPV. A higher level of husband’s education (OR ranging 0.490–0.854) and women having decision making agency in the household (OR ranging 0.897–0.835) were negatively associated with occurrence of IPV. On the other hand, having daughters (OR 1.094–1.427), rural residence (OR = 1.109), habit of smoking (OR = 1.133), tobacco (OR = 1.350) and alcohol consumption (OR = 1.194–2.053) are positively associated with perpetration of IPV by husbands. Husbands who are regular alcohol consumers are more than twice as likely to perpetrate violence on wives than those who do not consume alcohol at all. Every other religion group have lower odds of committing spousal violence than Hindus. Similarly, for caste categories, belonging to ST, OBC or other castes (OR ranging 0.792–0.913) have lower odds of committing IPV than men belonging to SC category. Comparing to the Northern region, all regions have higher odds of husbands committing IPV. Men who justify wife beating have twice has higher odds of committing IPV than those who do not have such attitude.

#### Model 3 economic characteristics

IPV was found to be negatively associated with men being employed as compared to unemployed men. Although the odds of committing IPV varies among different types of work. The increasing wealth is also negatively associated with husband committing IPV, lowest among the rich (OR = 0.531) households.

#### Model 4 final model (all variables)

Older men were found to be more likely to perpetrate spousal violence. Men who are over 50 years (OR = 1.977, 95%CI = 1.068–3.660) were 97% more likely to commit IPV as compared to married men below 20 years. Men in their 30s (OR = 1.923, 95%CI = 0.832–2.838) were also over 90% more likely to commit violence than the men under 20 years of age. Although age gap among husband and wife was found to be a protective factor. There is 30% to 40% less likelihood of men perpetrating violence against their wives in cases of larger age gap where the difference is 15–20 years or more than 20 years.

Indian husbands are less likely to inflict IPV as their educational level increases. As compared to men with no education, husbands who have primary (OR = 0.896, 95% CI = 0.830–0.968), secondary (OR = 0.747, 95% CI = 0.699–0.798) or higher (OR = 0.581, 95% CI = 0.525–0.643) education were about 40% to 10% less likely to indulge in IPV. Individual’s social background such as religion, caste and region of residence has a significant impact on their tendency to commit IPV. Christian men were 32% less likely to perpetrate violence as compared to Hindu men amongst various religious groups. Amongst caste categories, men belonging to Scheduled Tribe (OR = 0.869, 95%CI = 0.797–0.948), Other Backward Class (OR = 0.903, 95% CI = 0.849–0.959) and others (OR = 0.819, 95% CI = 0.883) are about 8% to 20% less likely to engage in IPV than men belonging to scheduled caste group. Men in Southern India (OR = 1.990, 95% CI = 1.785–2.218) have significantly high probability of committing IPV as they were found to be twice as likely to perpetrate spousal violence as compared to Northern region of India, followed by Eastern region (OR = 1.605, 95%CI = 1.442–1.787) and then Central India (OR = 1.521, 95%CI = 1.355–1.708) and North-eastern region (OR = 1.378, 95%CI = 1.192–1.593).

As compared to men who have no daughters, those who have 1 or 2 daughters (OR =, 1.079 95% CI = 1.025–1.136) are about 8% more likely to perpetrate violence whereas those with more than 2 daughters are about 35% more likely to commit violence on their wives. Wives decision making autonomy where the wife takes household and personal decisions alone or equally with husband negatively associates with IPV. It was found that there is about 15% less likelihood of men perpetrating violence where wives have high (OR = 0.861, 95% CI = 0.795–0.932) decision making agency. On the other hand, men’s IPV justifying attitude results in positive association with actual violence perpetration. Men who justify wife beating (OR = 1.181, 95% CI = 1.124–1.241) are about 20% more likely to commit IPV.

Men who indulge in substance abuse such as smoking (OR = 1.123, 95% CI = 1.051–1.200), consuming tobacco (OR = 1.312, 95% CI = 1.244–1.383) or those who consume alcohol on a regular (OR = 2.020, 95%CI = 1.822–2.239) or occasional (OR = 1.220, 95% CI = 1.153–1.291) basis are more likely to commit violence than those who don’t indulge in any kind of substance abuse. Regular drinkers or alcoholic men as more than twice as likely to commit violence than those who never drink.

Employed men who do mental and manual labor (OR = 0.654, 95%CI = 0.553–0.773), light manual labor (OR = 0.720, 95% CI = 0.619–0.837), and heavy manual labor (OR = 0.821, 95% CI = 0.710–0.950) are about 20% to 35% less likely to commit IPV than the unemployed men. The likelihood of men committing spousal violence gradually decreases as wealth of the household increases. Men belonging to middle income (OR = 0.919, 95% CI = 0.862–0.980) and rich (OR = 0.686, 95%CI = 0.641–0.734) families are about 10% to 30% less likely to commit IPV as compared to those who belong to poor households.

## Discussion

The current study aimed at locating the bio-demographic, social and economic characteristics of IPV indulging men in India. Firstly, one-third of all ever-married women reported have suffered at least one type of violence from their current husbands. The most prevalent domestic abuse perpetrated by husbands was physical violence (88%) followed by emotional and sexual abuse. Men’s indulgence in IPV is positively associated with their age. Men of older age were more violent towards their wives than the younger generation, suggesting a declining trend in domestic violence in India. We found several factors that are associated with increase or decrease in men’s indulgence in IPV. Through binary regression models, we were able to quantify the degree of association with these characteristics of men and their indulgence in IPV. Education, wealth, employment, wife having decision making autonomy were found to be protective factors whereas smoking, alcoholism, higher number of female children, wife beating attitude, and unemployment were found to be risk factors of men’s indulgence in IPV. Men’s social and religious background and regional location and place of residence (rural/urban) were also found to be significantly influence their IPV perpetrating behavior.

Substance abuse in form of smoking and alcohol consumption has been consistently highlighted as significant factors of violence against women in studies across the world, as was found in this study. A partner’s heavy alcohol consumption may increase stress and financial problems in a relationship contributing directly to marital conflict and the risk of IPV [5, 16, 39]. Alcoholic men are nearly three times more likely to commit IPV than non-alcoholic men [30]. Dependency on alcohol for heavy drinkers may culminate to lack of communication and result in lesser family support, leading to occurrence of conflicts within families [27]. Behavioural factors such as men’s IPV justifying attitude was found to be a prominent risk factor of their indulgence in IPV as found in many previous studies [25, 40, 41]. IPV justifying behavior has often been associated with childhood exposure to inter-parental violence and other socio-demographic factors [22, 23]. These factors might exist in combination and result in varied level of acceptance of and indulgence in IPV. For example, in a recent study in Bangladesh, it was found that men with low education and those who were alcoholic were considerably more likely to perpetrate IPV against their female partners [30].

Education has been associated with reducing the risks of IPV in many previous studies. Education generates positive changes in various dimensions associated with IPV in general, such as reducing wife-beating justification [23], awareness of women’s rights [4], increasing wife’s decision-making autonomy [42], and in effect reducing women’s experience of domestic violence [6, 27]. Similarly, we found that education reduces the risk of men’s indulgence in IPV. Consequently, husbands with little or no education were more likely to indulge in IPV [43–45], similar results have been obtained in the current study.

Unemployment and labour have implication towards increased stress and familial conflicts [46]. The findings of this study remain consistent with previous ones in finding that unemployed men having the highest odds of perpetrating violence against their wives. Unemployment and hard labor have been associated with increased risk of alcohol and substance abuse, which are, linked with indulgence in violence [47, 48]. On the other hand, poor financial conditions arising from unemployment and low paying laborious job can increase dissatisfaction and emotional stress resulting in aggression and violence on one’s partner [30]. Consequently, wealth was found to be a protective factor for men’s indulgence in IPV. It is argued that wealth becomes a protective factor indirectly through increased access to media and education [16], better living conditions and opportunities.

Apart from these individual characteristics, social factors such as son preference in Indian society is argued to be a significant predictor of violence against women [49]. We found that the chances of men’s indulgence in IPV increases with increasing number of daughters. Women are often, unscientifically, held responsible for the gender of the child, giving birth to a daughter is linked with increased risk of domestic violence. Consequently, resulting in a concerning manifestation of sexual and physical violence directed towards their wives. This behavior typically stems from a complex interplay of factors, including deep-seated frustration, a fear of social exclusion, and a desire to avoid societal shame [50].

Religion and caste are major determinants of individual’s financial, educational and social condition and overall human development in India. They are also major influencers in social and familial dynamics. Belonging to SC/ST categories have been linked with increased odds of poor educational attainment, lower paying laborious jobs, poor financial status and poor mental health, arising from long standing casteism and discrimination [51–53]. These factors have been previously associated with increased risk of men’s indulgence in IPV. Henceforth, we can associate the higher odds of SC/ST men’s indulgence in IPV as a resultant of complex social structures and persistence casteism in India.

Place of residence was found to be a significant predictor of men’s indulgence in IPV. Tranchant and Mueller [54] found that rural areas are more susceptible to an increased likelihood of IPV experience as women living in rural areas reported the highest beating [29]. However, as found in this study, men in both rural and urban areas are prone to commit IPV under different circumstances, though urban men were found to be committing lesser IPV than rural men, such as in Ghana where urban men associate with a lesser likelihood of committing IPV due to reduced stress and alcohol consumption, better employment opportunities, welfare accessibility, accommodating gender norms and expectation [28] as compared to rural men.

India, having distinct features in its various regions, reflects diversity in men’s attitude and behavior as well. We found significantly higher odds of men’s indulgence in IPV in southern region of India compared to the northern region, similar to some previous studies [35]. Southern states are characterized as better off states of India in terms of human development indicators such as overall health, education, financial and living situations, sex ratio, etc. [2]. Having such higher prevalence of men’s indulgence in IPV raises the question over the effectiveness of education, wealth and social determinants in reducing the prevalence of IPV. This particular finding of the present study underscores the intricate nature of determinants influencing IPV and emphasizes the necessity for additional research within the specific context under investigation.

Therefore, merely criminalizing the act of domestic violence might not be as effective in combating violence against women in India. The first step in addressing domestic violence against women was taken with the enactment of The Dowry Prohibition Act, 1961 (1984, 1986). Domestic.violence, including IPV, was criminalized in Section 498A of the Indian Panel Code ensuring that husbands and his relatives can face 3 years of imprisonment and/or a fine if found guilty [55]. On a similar note, Section 304B of the Indian Panel Code holds the husband and in-laws criminally responsible if a woman dies under suspicious circumstances within 7 years of marriage [56]. The Protection of Women from Domestic Violence Act, 2005 [57] is a policy put forward to combat the domestic violence against women in India allowing women to seek restraining orders against husbands/partners and creating criminal provisions of imprisonment and fines if orders are breached along with certain corrective measures. However, there are no such targeted provisions or policy intervention in place focusing on demographic, socio-economic and cultural characteristics of IPV committing men in India. Our study, in an attempt to shift the policy gaze from victims to perpetrators, comprehensively analyses husband’s characteristics and locates the perpetrators in their socio-economic and demographic context. The study, using a most recent, largest and reliable data set, successfully extracts a set of Indian men’ characteristics which can be seen as risk factors of IPV and pose a real threat to women’s health and well-being. We therefore suggest that the priority should be given to education, control on substance abuse and alcoholism among men, better employment opportunities, while putting major focus on poorest households. There is a need for strategic intervention to modify the factors associated with social acceptance of IPV in terms of male dominance, consent education to reduce sexual violence, and creating a safer space for women in both urban and rural areas to seek help.

## Strength and limitations

This study utilized a large-scale representative sample distributed well across the country, which enables us to generalize the findings at the national level. Our study uses the most recent and largest data available in India for understanding the underlying husband’s characteristics in relation to IPV. We analyzed husband’s characteristics using the responses of husbands themselves to avoid any bias and misinformation. Past studies in Indian context analyzing the characteristics of IPV committing husbands primarily focused on alcoholism and education. Unlike the existing studies, this research presents a fuller and comprehensive account of characteristics of IPV perpetrating husbands at all India level. Though it was not possible to assess a causal relationship in this study. A data-related limitation is that the data were based on self-reports, so the data may be subject to recall bias. Another data-related limitation of this study is that we have only used the cases of IPV committed by current husbands and excluded past husbands since we needed the responses for various background characteristics as answered by the men.

## Conclusion

The study attempted to locate the perpetrators of IPV among Indian men (husbands) in terms of their demographic and socio-economic characteristics. It unravels that IPV likelihood is directly related to a husband’s relative age, illiteracy, or lower level of education, smoking and other forms of tobacco consumption, regular alcohol consumption, and poor wealth index. It also finds spatial variations in terms of type of residence (rural versus urban) and across various regions of India. The results of the study show that samples, with no education, having more than 2 daughters, and belonging to the southern region of the country present a probable characterization of Indian husbands committing IPV. On the other hand, husbands with higher education level, employment in the organized sector, and positioned in higher wealth quintile positively minimize their chances of indulging in IPV. Thus, it is recommended that focused public policy intervention targeted to the macro level factors (aiming positive modification in Indian men characteristics) from the perspective of women safety health and wellbeing be enacted to address the root causes of intimate partner violence.

## Supporting information

S1 Table Description of selected husband’s characteristic variables. (DOCX)
