# Supplementary material for: Locating perpetrators of violence against women in India: An analysis of married men’s characteristics associated with intimate partner violence
Source: PLoS One. 2023 Aug 4;18(8):e0289596. doi: 10.1371/journal.pone.0289596 (PMC10403108; doi:10.1371/journal.pone.0289596)
Supplement: S1 Table — (DOCX) [file pone.0289596.s001.docx]

**Supplementary file**

**S1 Table.** **Description of selected husband’s characteristic variables.**

| **Independent variable** | **Description** | **Coding** |
| --- | --- | --- |
| ***Demographic*** | | |
| **Husband’s age** | Age of the ever married men respondents, which has been classified into 5 categories of teenage (15-19), twenties (20-29), thirties (30-39), forties (40-49) and more than 50 years old (>50). | 0= 15-19, 1= 20-29, 2= 30-39, 3= 40-49, 4= >50 |
| **Age gap** | Age gap between husband and wife has been calculated using the current age of husband and wife and categorized into groups of younger than wife, 0-5 years, 5-10 years, 10-15 years, 15-20 years and more than 20 years of age gap. | 0= Younger than wife, 1= 0-5, 2=5-10, 3= 10-15, 4=15-20, 5= >20 |
| **Abdominal obesity** | The waist to hip ratio in men to estimate health risks as prescribed by WHO and categorized as <.0.95 as low risk, 0.95-1.00 as moderate risk and >1.00 as high risk individuals | 0= Low risk  1= Moderate risk  2= High risk |
| ***Social*** | | |
| **Husband’s education** | It is the highest level of education obtained by men, classified in 4 categories | 0= No education, 1=Primary, 2=Secondary,  3= Higher |
| **Religion** | Religion of the husband | 1= Hindu, 2= Muslim, 3= Christian, 4= Sikh, 5= Other |
| **Caste** | Caste category of the husband | 1= Scheduled Caste (SC),  2= Scheduled Tribe (ST),  3= Other Backward Class (OBC), 4= Other |
| **Residence** | The place of residence has two categories; urban and rural | 1= Urban, 2= Rural |
| **Region** | Region of the country where the respondent is from. States and union territories are classified into 6 regions. | 1=Northern,  2= Central,  3= Eastern,  4= Western,  5= Southern,  6= North-Eastern |
| **Number of daughters** | Number of living daughters that the women gave birth to is classified into 3 categories: No daughters , 1-2 daughters, and more than 2 daughters. | 0 = 0, 1= 1-2  2= >2 |
|  |  |  |
| **Wife’s decision making autonomy** | It is a composite index of the responses to five household decision making related questions measuring the level of wife’s autonomy in decision making.   1. who do you think should have the greater say when making major household purchases 2. for daily household purchases 3. when deciding what to do with the wife’s earning 4. when deciding what to do with the respondent’s (men) own earning 5. when deciding how many children to have   if the decision is made by wife alone or equally with husband it is considered as “Yes” to decision making autonomy and if the decision is solely made by husband then a “No”. If the wife is making 0-1 household decisions = low autonomy, 2-3 decisions= medium autonomy and 4-5 decisions = high autonomy | 1= Low, 2= Medium, 3 = High |
| **IPV justifying attitude** | If the husband justifies wife beating in any of the seven cases-   1. Wife goes out without telling 2. Wife neglects the children 3. Wife argues with husband 4. Wife refuses to have sex with husband 5. Wife burns food 6. Wife is suspected to be unfaithful; 7. Wife disrespect in laws | 0 = No, 1 = Yes |
| **Smoking** | Smoking habit of the husband; includes cigarettes, cigar, pipe or hookah | 0= No, 1=Yes |
| **Consumes tobacco** | Means whether or not the husband chews tobacco or khaini | 0= No, 1=Yes |
| **Alcohol frequency** | Frequency of alcohol drinking by husband has been classified into 3 categories- none as never, every day or once a week as regularly and less than once a week as occasionally | 0= Never,  1= Regularly  2= Occasionally |
| ***Economic*** | | |
| **Labor type** | Occupation of the husband classified into 5 categories based on the type of work done by the husband at the job. | 0= Unemployed  1= Mental work  2= Mental & Manual labor  3= Light Manual labor  4= Heavy Manual labor |
| **Wealth index** | Economic status of the household is classified into 5 categories based on the composite index of the various household assets in the respondent’s house | 1= Poorest,2=Poorer3= Middle, 4= Richer, 5= Richest |
